# Recent Advances in Plant Somatic Embryogenesis: Where We Stand and Where to Go?

**DOI:** 10.3390/ijms25168912

**Published:** 2024-08-16

**Authors:** MªTeresa Martínez, Elena Corredoira

**Affiliations:** Misión Biológica de Galicia, Sede Santiago de Compostela, Consejo Superior de Investigaciones Científicas (MBG-CSIC), Avda Vigo s/n, 15705 Santiago de Compostela, Spain; temar@mbg.csic.es

Somatic embryogenesis (SE) is a fascinating example of the plant cellular totipotency concept [1,2,3]. During this regeneration process, one or more somatic cells are subjected to favorable in vitro experimental conditions and undergo morphological and biochemical changes that induce the formation of bipolar structures, known as somatic embryos [4]. Although gamete fusion does not occur, somatic embryos resemble zygotic embryos and have the capacity to germinate and develop into somatic seedlings [5]. In addition to their evident application in clonal propagation, somatic embryos represent excellent target material for the insertion of isolated gene sequences in somatic cells and the subsequent production of genetically modified plants [6]. This classic engineering technology is expected to be replaced by the emerging field of plant genome editing, which will probably require embryogenic cultures as a means of regenerating edited plants [7,8]. In addition, the application of synthetic seed technology to somatic embryos is being considered with regard to storage and handling [9]. Somatic embryogenic lines can be subjected to well-developed cryopreservation techniques for the long-term conservation of plant diversity or conservation during field testing of regenerated plants and subsequent retrieval of superior lines from liquid nitrogen (LN) for exploitation [10,11]. Finally, SE is an ideal system for studying the molecular mechanisms that regulate developmental plasticity in plants [12,13].

Many of these aspects of SE are examined in this Special Issue. Four of the papers report studies of different aspects of the induction step, with special emphasis on the molecular mechanisms involved in the acquisition of embryogenic cell competence. Understanding the molecular mechanisms underlying SE is essential for resolving the problems related to the low induction rate and the difficulty in inducing somatic embryos in recalcitrant species/genotypes or in explants derived from adult trees [13,14]. Thus, for example, Ref. [15] reviewed the various in vitro plant regeneration processes that can converge in callus induction and analyzed the role of plant growth regulators (PGRs) in diverting callus formation from normal development. Furthermore, this author summarized the available information on the 21 genes, which indicates the pre-meristematic nature underlying the broad plant regeneration potential of callus tissue.

During the induction of SE, cells must acquire a state of embryogenic competence, a process that is usually governed by hundreds of genes [16]. In this respect, Ref. [17] identified the *HD-ZIP* genes that play an important role in embryonic development across the entire genome of oil palm species. The total of 26 *EgHDZIP* genes identified was divided into four subfamilies based on similarities in gene structures and conserved protein motifs. The expression profiles are displayed during somatic and zygotic embryogenesis, and the expression patterns appear to be the opposite of those of *BABY BOOM* (*BBM*), a SE-marker gene. Thus, results revealed that *EgHDZIPIV* genes were up-regulated at the late stages of SE (torpedo and cotyledon), whereas the BBM gene was up-regulated at the globular stage of SE. These findings suggest that the manipulation of *EgHD-ZIPs* and *BBM* expression will facilitate and accelerate SE during oil palm tissue culture.

Transcriptional regulation is known to play an essential role in SE, with transcriptional initiation by transcription factors (TFs) being the principal means of regulating gene expression during the SE process [12,13,18]. In their review paper [19], the authors discuss the functions and role of the *WUSCHEL-RELATED HOMEOBOX* (*WOX*) transcription factors in inducing cell totipotency and in regulating SE, while also considering the interactions of WOX with other TFs, PGRs, and epigenetic signals. These authors conclude that *WOX* genes are involved in the in vitro regeneration of plants and, more generally, in the manifestation of cellular totipotency.

Among PGRs, auxins are considered key factors in SE induction [14]. Exogenous auxin treatment and endogenous auxin levels are determining factors in SE induction [20,21,22]. Following this idea, the action of auxin was the subject of research conducted by Ref. [23]. These authors studied the dynamics of endogenous auxin during in vivo gametophytic development relative to the profile during in vitro microspore reprogramming and embryogenesis induction and progression in *Brassica napus*. The study findings led these authors to infer that auxin plays a key role in the change in the developmental pathway of the microspore, where the opposite auxin dynamics determine the change in microspore cell fate, with auxin biosynthesis being required for induction of microspore embryogenesis and during subsequent embryo development. 

Another group of papers report different practical applications of SE. Somatic embryogenesis is, for instance, of great importance in forest management and breeding programs [24,25], which face the enormous limitation of the long generation time of tree species [26]. The application of SE enables faster generation of new improved genotypes through classic genetic transformation, genome editing tools, priming, or stress memory [27,28]. Priming is defined as an imprint left on plants when exposed to stress, after which they become more tolerant to future similar or different types of stress [28,29]. In this Special Issue, two papers describe the application of priming to embryogenic cultures to produce plants with enhanced tolerance to drought and heat stress. In the first paper by Ref. [30], the authors analyzed epigenetic changes in 3-year-old priming-derived plants of maritime pine (*Pinus pinaster*) growing in the greenhouse and subjected to 30-day drought stress. The authors demonstrated that high-temperature pulses during SE produce epigenetic marks in primed somatic plants and result in transcriptomic and physiological changes in plants. Such changes can increase plant resilience to drought stress since heat-primed plants exhibit permanent activation of cell protection mechanisms and overexpress stress pathways that pre-adapt them to respond more efficiently to soil water deficits. These changes consist of higher constitutive contents of osmoprotectant compounds such as proline, soluble proteins, glutathione, and starch in needles, as well as increased expression of genes coding for transcription factors involved in the early stress response of genes coding for proteins known to prevent cell damage and of genes coding for enzymes involved in an oxidative stress response. In the second paper, Ref. [31] found that applying heat stress to embryogenic cultures of *Pinus halepensis* during the SE induction phase led to proteomic and metabolomic reorganization. These authors observed that several enzymes directly involved in metabolic pathways and proteins previously found to be involved in temperature stress response, such as histones and ribosomal proteins, occurred in higher amounts at higher temperatures. These findings seem to reinforce the idea that a temperature priming effect during the initial stages of SE can trigger long-lasting effects in the mechanisms involved in heat stress response.

As mentioned above, genome editing is another way of generating improved genotypes. As in the classic genetic transformation, a procedure for regenerating plants from the modified cells is required [6], with plant regeneration from protoplasts being the preferred method [32]. In this respect, Ref. [33] investigated the production of protoplasts from embryogenic and non-embryogenic cells in carrots, a model plant. The results obtained showed that the protoplast isolation process and plant regeneration were more efficient when embryogenic cells were used as explants to establish cell suspension cultures. These authors also confirmed that the expression of specific genes, such as *SOMATIC EMBRYOGENESIS RECEPTOR-LIKE KINASE* (SERK), *WUSCHEL*, *BBM*, and *LEAFY COTYLEDON*, involved in the SE process were maintained after protoplast isolation.

Cryopreservation is a very useful procedure in SE, as it reduces the costs associated with maintaining embryogenic cultures [34,35]. However, the investment required for maintaining cryobanks and the continuous supply of LN greatly limit the use of this technique. The use of ultra-low temperature freezers (−80 °C) is a simple and economical alternative to LN for conserving embryogenic lines previously dehydrated to prevent cell damage, although this conservation technique has scarcely been used to date [36]. In this regard, Ref. [37] attempted to reduce the cost of previously defined cryopreservation procedures for conserving embryogenic cultures of *Picea abies* L. Karst. These authors first investigated whether it was possible to store embryogenic cultures of Norway spruce (originating from elite trees obtained in the Finnish tree breeding program) in freezers (at −80 °C) instead of in LN, concluding that frozen storage of embryogenic cultures at −80 °C was not feasible. However, they managed to simplify the previously used cryopreservation protocol by using only one pre-treatment and post-thawing medium with high sucrose content instead of two pre-treatment steps, thereby reducing the cost of the technique.

Finally, in a review article, Ref. [38] summarizes the advances in SE in banana, with special emphasis on the molecular basis underlying the process and on the potential application of this knowledge to optimize the induction step in recalcitrant banana cultivars.

In summary, all nine papers published in this Special Issue deal with very different aspects of SE, covering different plant species, including several woody species. The editors consider that all articles published in this Special Issue provide useful information on the molecular mechanisms underlying the acquisition of embryogenic competence and on the application of SE in plant breeding and conservation. Although SE is considered a powerful tool in plant biotechnology [39], interdisciplinary research and technological efforts must be continued in order to address the remaining challenges [40].
